# Self-reported motivation for choosing nursing studies: a self-determination theory perspective

**DOI:** 10.1186/s12909-019-1568-0

**Published:** 2019-06-10

**Authors:** Linda Messineo, Mario Allegra, Luciano Seta

**Affiliations:** Istituto per le Tecnologie Didattiche, Consiglio Nazionale delle Ricerche, via Ugo La Malfa 153, Palermo, 90146 Italy

**Keywords:** Autonomous motivation, Choosing nursing, Content analysis, Controlled motivation, Nursing students, Self-determination theory

## Abstract

**Background:**

The nursing shortage is of worldwide concern, with nursing student retention acknowledged as a priority. As a fundamental step towards exploring factors that can guide the implementation of strategic approaches to retain undergraduate nursing students and prevent their attrition, the aim of this study is to examine the motivation for choosing nursing studies of first-year nursing students within the theoretical framework of self-determination theory.

**Methods:**

We conducted a study at the Medical School of the University of Palermo. A total of 133 first-year nursing students completed a two-part questionnaire: a measure of socio-demographic aspects and an open question about their motivation for choosing nursing studies. Students’ responses were analysed using thematic analysis. Dimensional analysis was performed in order to verify an organization along one dimension, in agreement with the differentiation of the autonomous and controlled types of motivation of self-determination theory. A person-centred approach was utilised to define motivational profiles able to characterize clusters of students according to both quality and quantity of motivation.

**Results:**

A set of 18 categories was developed. The factor analysis has shown that nursing students’ motivations can be organized along one dimension, in alignment with the differentiation of the autonomous and controlled forms of motivation of self-determination theory. Through adoption of a person-centred approach, four motivational profiles were identified: a) students with good quality motivation profile (high autonomous and low controlled); b) students with poor quality motivation profile (low autonomous and high controlled); c) students with low quantity motivation profile (low autonomous and low controlled); d) students with low quantity and poor quality motivation profile (i.e. prevalence of controlled motivation).

**Conclusions:**

Importance of this research includes the possibility to interpret nursing students’ reasons within the theoretical framework of self-determination theory, a well-grounded model able to offer useful information to academic nursing schools, in order to promote effective strategies to foster and support student motivation.

## Background

Nursing is a profession of vital importance. Nurses play a significant role in the promotion of health, prevention of disease and care of the sick. The shortage of nurses in health systems is of worldwide concern [1, 2] and also poses a number of challenges to the educational system.

The nursing student attrition rate is one of the factors that negatively affects the satisfaction of health professional workforce demand. Planning approaches to promote the retention of undergraduate nursing students, especially during the first year, is a strategy to face the challenges of the shortfall of qualified nurses. The efficacy of these strategies requires better comprehension of the factors associated with nursing student attrition through fostering and supporting specific affective factors positively related to retention, and reducing modifiable factors positively related to attrition. Different levels and types of reasons and factors contributing to nursing students’ attrition have been identified [3–8]. Personal reasons for leaving, institutional, political, societal and occupational factors are reported [9]. Researchers have also examined how both students’ personal characteristics, such as study motivation, and environmental aspects influence their retention [4, 10–12]. Motivation is a construct that may influence student retention, academic achievement and persistence behaviours [11].

In the present study, the focus is on undergraduate student motivation in choosing nursing studies and the nursing profession. Comprehending the career motives for this choice is important in order to design specific strategies to promote undergraduate nursing student retention [13–15].

Motivation is a complex construct with multiple components. It is the result of a set of processes of activation and orientation of behaviour and action, aimed at the realization of a specific purpose. In the multifaceted process of choosing an academic degree course and a career, different sources, goals and reasons which drive intentional actions are present as co-occurring factors [14, 16]. Student reasons for choosing a nursing university course and nursing as a career have been explored in several research studies. These studies have shown that this choice is a result of a combination of different motivations. The most frequent reasons found in the literature are the wish to help other people, the desire to engage in activities and to perform a job which is socially beneficial, career opportunities and job security, the positive image of the specific role of nurses, the influence of family members or friends [17–23]. Personal experiences, such as hospitalization, illness of a family member, are identified as additional motivations for the choice [18–20]. In all research, help for others and altruism are the motivational factors for the career choice most frequently reported by students.

These motivations, due to their frequent occurrence, are often generic and their use can make it difficult to clearly understand the actual motivational process behind the student’s declaration. So, a similar term may refer to an internal process (such as a personal interest or internal pressure), or an external one (parental influence, peer pressure, situational factors, etc.). Any strategy that want to increase nursing students’ retention and set up efficient learning approaches, has to be based on a deeper understanding of their motivations. The use of structured questionnaires can help to avoid some inconsistencies. These tools are not very simple to implement, and the interpretation of the results requires a deep knowledge of the theory supporting their creation. Generally, the constructs are rigorously defined and the questions are strictly focused to measure only these factors. On the other hand, the use of open questions simplifies the gathering of self-reported motivations, and allows students to describe their beliefs and emotions about their choice of the degree course. However, it transfers to the researcher the demanding task of interpreting the students’ answers, and does not allow them to extend the results beyond the specific study.

This study illustrates how the theoretical framework of self-determination theory (SDT) [24, 25] can be used as a common framework for interpreting the self-reported reasons for choosing nursing studies, collected responding to an open question. This result will allow educationalists to construct shared strategies, once they have obtained a clear ‘motivational landscape’ characterizing the students of a particular degree course.

SDT is a theory of human motivation that hypothesizes that people pursue goal content through different regulatory processes. It identify three different categories of motivation that extend along a continuum of increasing self-determination: amotivation, controlled motivation and autonomous motivation [24]. Along this dimension, it is possible to distinguish specific types of regulatory styles, that vary in their degree of relative autonomy: from external to intrinsic regulation.

**Fig. 1:**
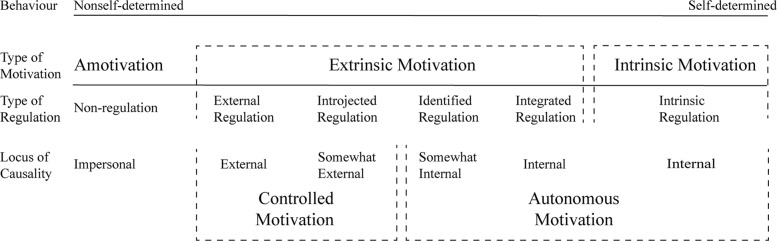
The self-determination continuum, showing the motivational, self-regulatory, and perceived locus of causality bases of behaviours that vary in the degree to which they are self-determined (adapted from Deci & Ryan, 2000)

The self-determination continuum, showing the motivational, self-regulatory, and perceived locus of causality bases of behaviours that vary in the degree to which they are self-determined, is illustrated in Fig. 1.

*Amotivation* is the state of lacking an intention to act [24, 25]. Amotivated individuals are neither intrinsically motivated nor extrinsically motivated [26].

*Extrinsic motivation* can vary greatly in the degree to which it is autonomous and it refers to pursuing a goal for reasons that are external to the activity itself. Considering the amount of relative autonomy, four types of regulation are recognized, namely *external regulation*, *introjected regulation*, *identified regulation* and *integrated regulation* [24, 25]. The continuum of motivation varies in term of the extent to which the objective of a behaviour has been internalized. *Externally regulated motivation* is the classic case of extrinsic motivation. It is low in self-determination, and the origin of individual’ behaviours is a particular external contingency, such as the achievement of an external reward that is considered to be positive, or the avoidance of a consequence perceived as negative. The control of goal-directed activity is entirely external. *Introjected regulation* refers to people that pursue goal directed behaviours for specific contingencies and consequences administered by individuals to themselves. Introjected regulation has been in part internalized, but it is a relatively instable form of regulation [24, 26]. External regulation and introjected regulation can be classified as *controlled motivation*.

*Identified regulation* is a more autonomous form of extrinsic motivation. Identification is a process through which people comprehend the value of an activity, and regulation is more internalized. The control is internal and the activity is sustained by self-regulation, and the objective has a value for the person.

*Integrated regulation* is the most autonomous form of extrinsic motivation. Integration is the process through which people perform an activity, integrating the identification with some aspect of the self. The control and the regulation are internal. Individuals feel competent when they perform an activity, and consider the activity as meaningful. An integrated regulation motivational state shares more characteristics with intrinsic motivation. Nevertheless, the activity is carried out to obtain a goal or outcomes that are different from the activity itself.

Finally, *intrinsic motivation* is a prototype of self-determined choice and activity. When intrinsically motivated, individual pursue a goal because of the inherent feeling of pleasure, enjoyment and satisfaction in the activity. The purpose corresponds with the activity itself. SDT suggests that people are intrinsically motivated to do something when they have the opportunity to autonomously choose meaningful activities that support their self-expression and that allow them to use their competence.

Identified and integrated regulation can be classified as *well-internalized motivations*; well-internalized and intrinsic motivations are the two subcomponents of *autonomous motivation*.

Many research studies have investigated the relationship between motivation and learning. Student motivation influences learning, study behaviour and achievement, and it is related to academic success [27]. Research shows that *autonomous motivation profiles* are associated to more successful learning outcomes and success, over time, than *controlled motivation* [28, 29]. Moreover, *autonomous motivation* is associated to the use of deep cognitive strategy and reflection in learning [30, 31]. Intrinsically motivated students persist much more in an academic task [31, 32]. Students with an autonomous profile, referred to high levels of autonomous motivation and low levels of controlled motivation, perform optimal learning patterns [33], and they are more persistent in an academic program than students with other motivational profiles [34]. Besides, intrinsic motivation may prevent academic disengagement [29].

Consequently, to understand the quality of personal motivation, in terms of *controlled* vs. *autonomous*, is a preliminary objective for any interventions aimed at reducing attrition and improving academic success.

The aim of the present study was to identify, using a qualitative research approach, representative categories able to summarise the content of first-year students’ self-reported motivations for choosing nursing studies. The categories were analysed within the theoretical framework of the self-determination theory, in order to verify their organization along a continuum scale, ranging from controlled to autonomous motivation.

Although previous qualitative studies that investigate self-reported motivation for choosing nursing studies are different, they have not usually examined students’ motivational statements from an SDT perspective. In a research study the statements on motivations of applicants for medical studies were analysed within an SDT framework [35]. This study shows that reasons of applicants mainly concerned autonomous motivations.

## Methods

### Methodological orientation and theory

In this study, a methodology is proposed to overcome some of the biases typical of self-report data. The qualitative analysis of the nursing students’ answers was integrated with multi-dimensional scaling techniques aimed at interpreting the results of the qualitative analysis in the theory-grounded framework of SDT.

The student nurses’ answers have been elaborated using qualitative and quantitative methods. The qualitative analysis was aimed at interpreting an abundant variety of personal information about career and study choices, instead of using defined tests in career choices, in which students have to select from limited answer options. The outcome of this analysis was a group of categories summarizing the main identified reasons.

The quantitative analysis used the SDT framework to organize the categories along the dimension controlled/autonomous. This allowed a comparison between the results of this study and other studies that used scales to measure student nurse motivation, giving external confirmation of the validity of the proposed interpretation.

### Participants and procedure

The study was conducted at the School of Medicine of the University of Palermo. According to the local ethical policy, no prior formal authorization approval by the Ethics Committee was necessary. We communicated the qualitative study design to the Institutional board of the Undergraduate Nursing Course guaranteeing that ethical standards would be met and we received approval.

Data collection was carried out on entry to the nursing degree course, at the beginning of the first month of the first semester. All undergraduate students (*N*_*tot*_=140) enrolled in the first year of the nursing degree were invited to participate in the study. One researcher (LM), a general psychology teacher in the nursing degree course, invited students attending her lectures to participate in the study.

After a general psychology teaching session, LM communicated the following information: (a) the aim of the research was illustrated; (b) participation was voluntary, and students could decide to interrupt their participation at any moment; (c) the students were reassured about the confidentiality of the data collected, which was only managed by researchers; (d) each name was replaced with a unique code number.

During the same session, LM presented the procedure for taking part in the study. Data was collected by filling in an on-line questionnaire by means of a web-platform structured for the purposes of the present research. Data was retrieved only after obtaining voluntary informed consent, in the presence of LM. The duration of the entire session was about 30 minutes.

### Measures

The questionnaire consisted in a two-part form: a) demographic survey aimed at gathering some demographic data about the students; b) motivation for choosing nursing studies.

*Demographic survey.* The demographic questions were concerned with basic demographic information such as age and sex. Students were also asked if they had previously attended other university courses and if so, specify the name of the degree course.

*Motivation for choosing nursing studies.* An open question was asked to gather information about student motivation regarding their decision to enrol on the university nursing course. Students had to answer the question: *“Describe your reasons for choosing the nursing degree course”*.

## Data analyses and results

The number of first-year nursing students who participated in the study and answered all questions was *N*=133. Seven students chose to not participate in the research. Participants included a total of 84 females (63%) and 49 males (37%), aged from 18 to 41 (*M*_*age*_=21.7 years, *SD*=4.31). Demographic data of student participants, regarding gender and age distribution, are reported in Table 1.

**Table 1:** Demographic characteristics of nursing students

Characteristics	n	%	Age: Mean (SD)
*Gender*			
Males	49	37	22.22 (4.72)
Females	84	63	21.32 (4.04)
Total	133	100	21.70 (4.31)
*Age groups*			
18-19	50	38	18.80 (0.40)
20-21	43	32	20.26 (0.44)
22-41	40	30	26.73 (4.85)
*Previous university experience*			
Healthcare sector courses			
or health-related sectors	26	19	22.50 (4.62)
Humanistic disciplines	9	7	27.11 (6.01)
Scientific subjects	13	10	23.77 (5.15)
No previous university experience	74	56	20.35 (2.96)
Non-responses	11	8	20.18 (2.36)

Thirty-six percent of the students had previously attended other university courses. Previous university student enrolments mainly consisted of attending healthcare sector courses, like pharmacy and psychiatric rehabilitation techniques (9%) or health-related sectors such as biology, biotechnology and motor science university courses (10%). Seven percent of the students had previously enrolled in humanistic disciplines, such as psychology and philosophy. The others students (10%) attended courses in scientific subjects, such as engineering, economics and information technology.

### Thematic analysis

The students’ answers to the open question were analysed using qualitative methods [36]. Two independent evaluators (the first and third authors of this study) scrutinised and coded the content of the open questions. LM is a psychologist and researcher, an expert in cognitive and clinical psychology, conducting research on education and motivation, and is familiar with the literature on SDT. LS is a PhD researcher, an expert in applied mathematics, data analysis and modelling, and is familiar with the literature on cognitive sciences. The process of category selection followed four phases. In the first phase, after an in depth reading of students’ responses, the two evaluators independently carried out an analytic segmentation of the contents, with the aim of identify distinct *nuclei* of meaning. To describe the content, they suggested some categories. In this phase, each evaluator added a brief explanation to the category and some phrases extracted from the students’ answers, to highlight the meaning of the category. In the second phase, the two evaluators compared their categories in order to select a common bundle. During the phase three, the evaluators read all the answers again independently, selecting one or more among the categories previously defined for each subject. On average three categories were selected for each subject (*M*=2.82,*SD*=1.17). Finally, the two evaluators met to compare their selection. The few discrepancies revealed were resolved through discussion. An initial set of 18 categories was developed. Tables 2 and 3 summarise the results of thematic analysis with a description of the inferred categories.

**Table 2:** Inductively inferred categories

Category	Code	Brief description	Characteristic phrase (code number)
Be useful	USE	The desire to feel useful	*“The wish to be useful to others.” (264)*
			*“I like to be useful and to help people.” (70)*
			*“The desire to be helpful to others.” (106)*
Curiosity	CUR	The desire of knowledge	*“I chose this degree course because I have always been fascinated by the world of health care.” (36)*
			*“I chose a degree course that would guarantee me a more immediate professional future …in addition to this motivation, another factor comes into play, even more important, namely the desire for knowledge.” (105)*
Family tradition	FAM	Family tradition: one or more components of the family is a healthcare professional, a nurse, a doctor, etc.	*“I chose the nursing degree course for various reasons. First of all, because I have relatives in the medical field who have passed on the desire to help suffering people.” (157)*
			*“I have relatives that are nurses and doctors; they introduced me to this world.” (149)*
			*“I grew up in an environment full of doctors.” (286)*
			*“…my motives are also family based, in fact my father is a nurse and I would be happy to follow in his footsteps.” (110)*
Gratification	GRA	To consider nursing profession a rewarding profession.	*“I consider nursing a rewarding field, in fact a series of actions to improve the patient’s condition of health are carried out! I consider nursing a rewarding profession!” (9)*
			*“I think I would feel gratified at the end of the day knowing that I helped.” (122)*
			*“It* (this work) *is something that gratifies me, that makes me feel good, that satisfies me.” (202)*
			*“I think that helping people who are ill and who are in difficulty is one of the most satisfying things that gratifies them.” (18)*
Helping others	HEL	The desire to help others.	*“I think that helping sick people is absolutely one of the most rewarding things, which gratifies you.” (18)*
			*“I want to take care of others.” (168)*
			*“The idea of being able to help people really appeals to me.” (266)*
Human contact	HUM	Seeking human contact.	*“I really love being in close contact with people.” (188)*
			*“I like to have personal contact with people.” (182)*
			*“I love to interact with people.” (258)*
Mission	MIS	To consider nursing profession as a mission.	*“Ensuring that people who suffer are able to find not only a good nurse, but also an angel of consolation. We have to get to know them and to serve them with love.” (131)*
			*“Helping people suffering is a mission to be pursued.” (36)*
			*“I’ve always wanted to make a contribution to society, helping others because a nurse’s job is really a mission.” (150)*
Personal experience	EXP	Personal experience, such as job experience (e.g. volunteering), hospitalization, a relative with an illness, etc.	*“The motivation that has driven me to this decision was the death of my grandparents (death due to illness), and the death of a special good friend, who died long ago, at a much too early age.” (36)*
			*“I had a painful personal experience, therefore I realized that my pathway was to help people.” (64)*
			*“The choice was reinforced also by many personal ‘negative’ experiences in hospitals during the last few years of my life.” (44)*
			*“This desire began after an event that occurred in my family.” (43)*
Personality	MIR	Skills, abilities or personal characteristics that students think they possess.	*“In addition to the skill acquired by study and practice, it need a strong sense of humanity, with patience, kindness and firmness. I think I have these personal characteristics.” (65)*
			*“I am a person who can listen and help …I think anyone who wants to undertake this type of profession should have this kind of personality.” (35)*
			*“Helping others has always been in my nature.” (187)*
Practical	PRA	Oriented to manual and practical applications	*“Helping those in need …not from behind a desk, but doing something concretely, with a hands-on approach.” (191)*
			*“I like the curriculum which includes not only theoretical topics but also practical aspects.” (184)*
			*“In this degree course, we will be immediately brought into contact with the concrete work, we will put into practice what we study.” (221)*
			*“It allows me to do something concrete to help others.” (251)*
Recommendation	REC	Recommendations of family members and friends.	*“…thanks to different orientation services of the university and also after talking with friends, I realized that my future would be right in this course of study.” (158)*
			*“My girlfriend is in the third year* (of nursing degree course) *and she inspired me a lot.” (182)*
			*“Friends also influenced me to make this choice.” (230)*
Role of the nurse	ROL	Attraction of the image of the nursing role.	*“I have always been attracted by the figure of the nurse as a person that is in direct contact with people and therefore has a very crucial role of responsibility.” (1)*
			*“I have chosen a nursing degree course because I think the nurse is one of the most noble and important jobs in the world of work.” (94)*
Second choice	SCH	The choice of a nursing degree course isn’t the first option (for example, students weren’t able to pass admission tests in other preferred degree courses).	*“I am a first year student at this university course, after I obtained a university degree in philosophy. Clearly, this is not a choice motivated by passion, because philosophy was my first choice, but motivated by the desire to find a future career.” (230)*
			*“…because last year, I wasn’t admitted to a physiotherapy degree course.” (163)*
			*“I wasn’t admitted to a medical degree course, then I choose this degree program.” (222)*
			*“Nursing is my second choice. In fact, I will would like to attend the course of medicine.” (258)*
			*“Nursing was my eighth choice.” (75)*
Secure Job	JOB	Job opportunities.	*“I made the decision to choose a course of study that would guarantee me a job opportunity.” (105)*
			*“I was driven by the possibilities of employment at European level.” (182)*
			*“This degree course offers many opportunities of employment.” (198)*
			*“The first* (motivation) *concerns job opportunities, in fact, the percentage of graduates who find a job after the first year is very high.” (188)*
Smile	SMI	People’s smiles are rewarding.	*“Helping others and seeing them happy would be gratifying for me, because I would have contributed to their lives.” (44)*
			*“…give a smile to people who need it.” (198)*
			*“I love the smiles of people.” (288)*
			*“Being useful to others, to give them a smile to relieve their suffering,…, is something that would make me completely satisfied.” (161)*
Topics	TOP	Interest in science subjects.	*“The main reason why I enrolled in this degree course is because I have a preference for scientific topics, such as biology and chemistry.” (109)*
			*“I have a great passion for healthcare and science subjects such as biology, anatomy, etc.” (49)*
			*“Interest in topics concerning the health sector.” (87)*
Vocation	VOC	The intention or desire to be a nurse is present from many years.	*“From my early childhood, I felt that the role of this profession was right for me.” (139)*
			*“I always wanted to be a nurse.” (152)*
Well targeted	TAR	The choice is motivated by a specific career goal.	*“When I think about my future, I see myself in the operating room assisting a surgeon.” (165)*
			*“After I graduate, I will be able to specialize in obstetrics.”(126)*
			*“I enrolled in this course because my ambition is to become a scrub nurse in the operating room.” (2)*

**Table 3:** Summary of students’ responses using categories described in Table 2

Code	*n*_*i*_	*n*_*i*_/*N*	Age_*i*_	*F*_*i*_/*n*_*i*_
HEL	78	.59	20.7	.64
JOB	44	.33	22.9	.48
ROL	31	.23	21.6	.68
SCH	27	.20	23.0	.59
TOP	27	.20	21.1	.78
VOC	25	.19	22.4	.72
EXP	23	.17	21.2	.70
MIR	21	.16	21.3	.71
HUM	19	.14	19.7	.68
USE	18	.14	20.4	.78
FAM	11	.08	21.7	.55
PRA	10	.08	19.7	.70
REC	9	.07	23.1	.56
TAR	9	.07	20.6	.78
GRA	9	.07	20.6	.67
SMI	7	.05	19.3	1.0
CUR	4	.03	24.5	.25
MIS	3	.02	20.7	.67

*In columns*: *n*_*i*_, absolute frequency of each category; *n*_*i*_/*N* (*N*=133 sample size), relative frequency of each category; *Age*_*i*_, mean age for each category, 21.7 (SD =4.3) mean (SD) age of sample; *F*_*i*_/*n*_*i*_, females for category (females in the sample 63%).

As described in Table 3, the most frequent reasons expressed by students are the desire to help others (HEL), often associated with the desire to being useful (USE) and have contact with other people in a healthcare setting (HUM). These students often consider their choice like a mission (MIS) or a vocation (VOC). The second most frequent motivation is regarding job opportunities (JOB). This type of motivation, probably connected to the current socio-economic situation, reveals the increasing demand for a nursing job, in the face of an increasing demand for healthcare services. The attraction of the image of the nursing role (ROL) is often associated with rewarding aspect of the profession (GRA and SMI). Students described their personal interest in the topics (TOP). Personal experience (EXP) such as job experience (e.g. volunteering), or hospitalization, having a relative with an illness, etc., are described by students. Some students referred that their skills, abilities or personal characteristics they think to possess are essential for the nursing profession (MIR). Role model influence was another typology of driving motivation: family tradition (FAM) and recommendations of family members and friends (REC) were described. It is also important to highlight that the choice of a nursing degree course isn’t always the first option (SCH), for example, some students weren’t able to pass admission tests in other preferred degree courses.

When one compares the frequencies of the different categories with respect to gender and age, some differences are apparent (see Table 3). In females’ replies are recurrent the categories that refer to the feeling of usefulness (USE, HEL, VOC, HUM, MIS), rewarded with a smile (SMI, GRA), or to the pursuit of specific career goals, such as specialisation in midwifery (TAR). In males’ answers the prevailing motivations are more oriented to the search for social security, status (JOB), or knowledge-wish fulfilment (CUR). Taking age into consideration, the younger students appear more oriented toward categories related to personal fulfilment, such as seeking human contact (HUM) and an engagement in activities with applicative implications (PRA). The older students seem to give more weight to motivations attributable to the search for social status and security, such as looking for new job opportunities (JOB), or following friends/parents’ recommendations (REC).

### Dimensional analysis

Although the results of the qualitative analysis are in good agreement with the research literature about the choice of the nursing degree course, without a theoretical framework, these agreement could sound purely anecdotal. A first look at the whole corpus of answers suggests good homogeneity in the motivations expressed by the students, after considering individual linguistic and communicative differences. According to the SDT approach [24], a latent dimension seems to emerge from their answers, along which the subjects can be distributed along a continuum ranging from controlled to autonomous motivation. To confirm this hypothesis, the first step was to verify the possibility of organizing the obtained categories, described in Table 2, along one latent dimension, corresponding to controlled/autonomous in the SDT approach. The factor analysis (FA) is the dimension reduction method elective in these cases. For this dichotomous data, literature suggests to work with a tetrachoric correlation matrix [37]. The construction of this matrix starts from the matrix G of the co-occurrences of the *m* observed categories, *C*_*i*_, with *i*=1,...,*m*. The element *G*_*ij*_ of the matrix G (see lower triangle in Table 4) represents the number of subjects in which the categories *C*_*i*_ and *C*_*j*_ are both present. The tetrachoric correlation was applied using these values (see upper triangle in Table 4).

**Table 4:** Co-occurences and tetrachoric correlation matrices

	HEL	JOB	ROL	SCH	TOP	VOC	EXP	MIR	HUM	USE	FAM	PRA	REC	TAR	GRA	SMI	CUR	MIS
HEL	78	-.198	-.168	-.124	-.368	.031	.364	.067	.337	.053	-.045	-.373	-.162	.119	-.015	.511	-.353	0.53
JOB	22	44	-.196	.100	-.132	-.090	-.388	.001	-.209	.195	.041	-.035	.520	-.150	-.146	-.420	-.100	-.248
ROL	15	7	31	-.101	.001	.123	-.190	-.073	-.284	-.097	.083	.253	.000	-.345	-.166	-.154	.053	.361
SCH	14	10	5	27	-.038	-.005	-.066	-.149	.130	-.387	.114	-.211	.567	.206	-.180	-.275	.066	-.094
TOP	10	7	6	5	27	-.213	-.451	-.022	.128	.057	-.036	-.002	.037	-.173	.056	-.110	.080	-.124
VOC	15	7	7	5	3	25	.086	.120	-.069	-.180	.302	.342	-.155	-.321	-.311	-.086	-.135	-.101
EXP	18	3	3	4	1	5	23	.168	.300	-.132	-.344	-.334	-.287	.119	-.089	.362	.141	.151
MIR	13	7	4	3	4	5	5	21	.340	-.306	-.160	.086	-.091	.134	-.263	.206	-.084	-.057
HUM	15	4	2	5	5	3	6	6	19	-.089	-.140	-.289	-.082	-.257	.157	.009	-.073	-.005
USE	11	8	3	1	4	2	2	1	2	18	.116	.144	-.049	-.036	-.022	-.187	-.038	.229
FAM	6	4	3	3	2	4	0	1	1	2	11	.303	-.101	-.110	-.101	-.076	.077	.096
PRA	3	3	4	1	2	4	0	2	0	2	2	10	.104	-.098	-.094	-.034	.088	.142
REC	4	7	2	6	2	1	0	1	1	1	0	1	9	-.056	-.047	-.044	.129	.126
TAR	6	2	0	3	1	0	2	2	0	1	0	0	0	9	-.037	.210	.129	.119
GRA	5	2	1	1	2	0	1	0	2	1	0	0	0	0	9	.201	.139	.362
SMI	7	0	1	0	1	1	3	2	1	0	0	0	0	1	1	7	.156	.235
CUR	1	1	1	1	1	0	1	0	0	0	0	0	0	0	0	0	4	.574
MIS	2	0	2	0	0	0	1	0	0	1	0	0	0	0	1	0	1	3

*Lower diagonal*: the co-occurrences *G*_*ij*_ of categories in students’ answers, the terms on diagonal correspond to *n*_*i*_. *Upper diagonal*: tetrachoric correlation between two categories.

The sample size (*N*=133) and the number of the selected categories (*m*=18) limit the validity of the FA. Some authors have suggested a sample size of at least 200 subjects and ratios of sample size to categories between 5 to 20 [38–41]. Further studies have highlighted that these requirements are dependent on specific characteristics of the data, such as the magnitude of the communalities, magnitudes of the loadings, the number of factors, and the number of variable for factors [42]. The data obtained in this study are near the lower limit, established by the standards, to obtain a significant estimation for the parameters of the factorial model. To better understand the validity of the FA results, the Kaiser-Meyer-Olkin Measure of Sampling Adequacy (KMO MSA) was calculated, a well-known indicator of the common variance within a data set [43, 44]. Nine categories were above the threshold of 0.5 (*MSA*_*i*_>.5) (in bold in Table 5).

**Table 5:** Results of the test of Kaiser-Meyer-Olkin MSA_*i*_ (*in bold the categories over the threshold* 0.5)

**HEL**	**JOB**	ROL	SCH	TOP	**VOC**	**EXP**	MIR	**HUM**	USE	**FAM**	**PRA**	**REC**	TAR	GRA	**SMI**	CUR	MIS
**.57**	**.51**	.45	.46	.43	**.55**	**.61**	.49	**.50**	0.45	**.53**	**.60**	**.50**	.41	.38	**.61**	.42	.47

In Table 6 the results of the FA are summarised. This result was obtained by means of a factor analysis, with one latent variable, and an oblique factor with a minimizing criterion.

**Table 6:** Factor loadings for the FA (*in bold the categories with absolute value of the factor loadings greater than*.35)

	**HEL**	**JOB**	ROL	SCH	TOP	VOC	**EXP**	**MIR**	**HUM**	USE	**FAM**	**PRA**	**REC**	TAR	GRA	**SMI**	CUR	MIS
Loadings	**.686**	**-.537**	-.307	-.204	-.297	-.107	**.769**	**.360**	**.452**	-.192	**-.355**	**-.452**	**-.417**	.294	.182	**.648**	-.026	.134

Ordering the categories according to the increasing values of the factor loadings, it is possible to interpret the result as a distribution of the categories along the latent dimension. Table 7 shows the results of this ordering. Considering the meaning of the categories summarised in Table 2, this pattern is in agreement with an interpretation of the latent dimension evidenced by the SDT approach. The distribution of the categories seems to reproduce a classification in which on the left-hand side there are categories related to a controlled motivation, while moving to the right there are more autonomous categories. In fact, the controlled motivations are externally regulated, that is typical of subjects with an external perceived locus of causality. In the types of controlled motivation, the search for a job (JOB), family and social recommendation (FAM and REC) are included. It is also possible to include motivations that are more internal, but not sufficiently internalized, because they are not related to the specific activities that the students will go on to do (PRA and TOP). Categories strictly linked to primary needs, such as relatedness (HEL and HUM), competence (EXP), and autonomy (SMI and MIR) belong to the type of autonomous motivations. It is also interesting to note that the more extreme categories, in bold in Table 7, are also the categories with values above the threshold of 0.5 in the KMO MSA test (see Table 5), with the exception of the category VOC.

**Table 7:** Ordering of categories using the outcomes in Table 6 (*in bold the categories used in the* CFA)

controlled	**JOB**	**PRA**	**REC**	**FAM**	ROL	TOP	SCH	USE	VOC	CUR	MIS	GRA	TAR	MIR	**HUM**	**SMI**	**HEL**	**EXP**	autonomous

This led us to perform a confirmatory factor analysis (CFA) using only 8 categories, the categories best able to identify the quality of the motivation. From the types of controlled motivation, we used the categories JOB, REC, PRA, and FAM. We used the categories EXP, HUM, HEL, and SMI for the types of autonomous motivation.

For this analysis, a logistic model with 2 parameters was applied. This model is particularly appropriate for analysing binary data [45]. According to the unidimensional Item Response Theory (IRT), using a similar model with two parameters permits to obtain two different estimations for each category: the simplicity of the selection (intercept) and the ability of each category to discriminate the motivational profile of the students (slope). The results of the model are summarised in Table 8. The value of the intercept is correlated with the frequency of each category in the students’ answers. The slope is more interesting. The sign of the slope divides the category into two types of motivations, the categories with negative slopes corresponding to the controlled motivation (JOB, FAM, PRA and REC); the categories with positive values of the slope correspond to the autonomous motivation (HEL, EXP, HUM and SMI).

**Table 8:** Parameters of the logistic model (in bold negative slopes = controlled motivations)

Category	Intercept (se)	Slope (se)
HEL	.426 (.251)	1.005 (.463)
**JOB**	-.883 (.333)	**-1.118** (.563)
EXP	-2.341 (.382)	1.763 (.947)
HUM	-1.933 (1.199)	.775 (.425)
**FAM**	-2.513 (4.622)	**-.511** (.519)
**PRA**	-2.921 (1.627)	**-1.024** (.770)
**REC**	-3.610 (.945)	**-1.646** (1.274)
SMI	-4.426 (.731)	2.040 (1.518)

The dependent variable of the logistic model measures the level of internalization of student motivation, in relation to the decision to attend the specific degree course. For each student, the detection of a category with negative slope moves her/him toward a poorer motivational profile, and the detection of a category with a positive slope moves her/him toward a richer profile. Using the estimations of the model, a person-centred analysis can be carried out. This analysis allows for the assignment of a motivational score to each student, according to the pattern of categories extracted from her/his answer.

**Fig. 2:**
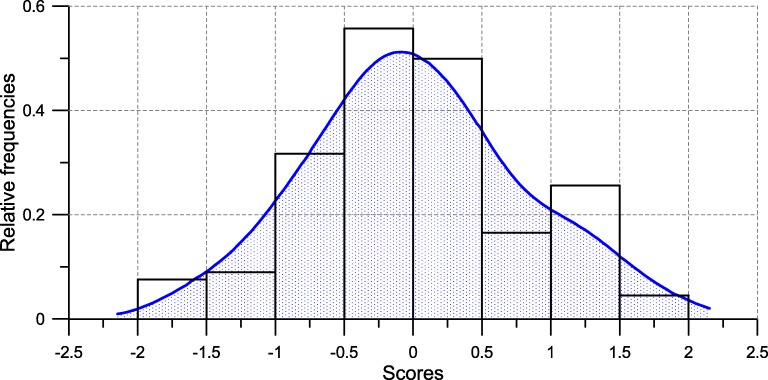
Distribution of the motivational scores

The distribution of these scores has a mean about zero (*M*=0.015,*SD*=.756). The test of Shapiro-Wilk makes it possible to reject the hypothesis of normality (*W*=.972,*p*<.01). The estimated value of the skewness, *g*_1_=.201(*se*=.212), highlights the fact that the mass of the distribution is concentrated on the left, i.e. on the controlled types of motivation (see Fig. 2).

**Fig. 3:**
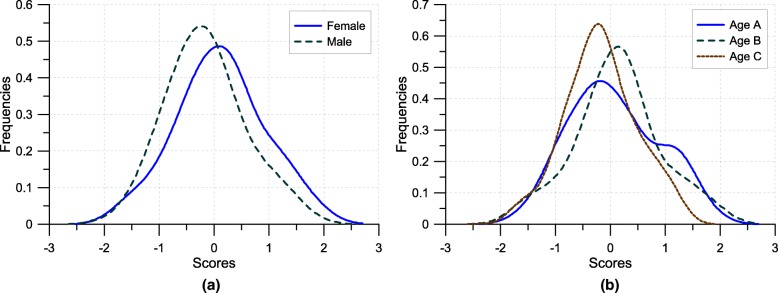
Scores for gender (**a**) and age (**b**) groups

Analysing the differences in the scores distribution for gender and age groups reveals some interesting differences. The means are different for females and males (*t*=2.074,*p*<.05), with males more controlled with respect to females. A comparison of the density distribution for gender groups shows they are not equivalent (*p*<.05). The visual analysis of the two distributions highlights that the males are more concentrated around the mean value (see Fig. 3a), although the Fligner-Killeen test does not permit to exclude the homogeneity of variances (*F*(1,131)=2.288,*p*=.23).

From our sample, we extract three age groups with comparable sizes: group A includes students aged between 18-19 years old (*n*_*A*_=50); group B students aged 20-21 (*n*_*B*_=43); group C students over 21 (*n*_*C*_=40). In group A there are novices, i.e. students who have recently graduated. Group B consists of students who obtained their high school degree at least one year ago, in some cases with previous experiences in other degree courses. Group C is more heterogeneous, frequently the members have had previous academic experience. The mean values for the groups, from A to C, are.057 (.792),.107 (.759), and −.186 (.620) respectively (standard deviations in parenthesis). The differences are not statistically significant (*F*(2.130)=1.901,*p*=.15). A visual inspection of the density distributions (see Fig. 3b) highlights some differences which are not significant (*p*>.2), with more variability in the scores distribution in the younger with respect to older students. Group C shows more controlled and homogeneous scores.

### Person-centred analysis

Using the results of the dimensional analysis, we classified subjects into groups with similar motivational profiles and constructed clusters of subjects who are characterised by similar combinations of motivations. The person-centred analysis, according to the study of Vansteenkiste (2009), permits also to verify the external validity of the results of this study. In the study [33], the authors have adopted a modified version of the *Self-Regulation Scale* (SR-S) [46]. The authors associated three standardized scores to each subject in the sample, which were calculated from the answers to the modified SR-S. These scores describe the quality of the motivation, controlled vs. autonomous, and the quantity of motivation, related to the total score in the questionnaire. To compare our outcomes, we used the result of the two-parameter logistic model to construct two variables to describe the quality of motivation for each student. The occurrence of categories JOB, REC, PRA, and FAM, permits a controlled motivation score to be allotted to each student, and the occurrence of categories EXP, HUM, HEL, and SMI, provides a score for autonomous motivation. The occurrence of each category is weighted using the estimated slope obtained in the model. To measure the quantity of motivation we counted the total number of categories identified by evaluators.

**Fig. 4:**
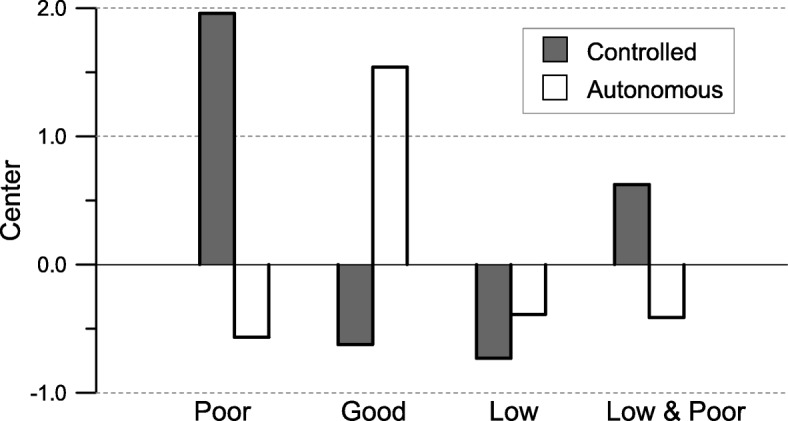
Motivational clusters

These three variables can describe the level of controlled and autonomous motivation for each student, and the total quantity of motivations in her/his answers. To obtain comparable results, a procedure of complete-linkage clustering was carried out. The optimal solution is composed of 4 clusters, with 68% of explained variance, and this result accords well with Vansteenkiste et al. (2009). Comparing the single cluster, some minor differences appear (see Fig. 4). We observe three clusters that are comparable with the clusters obtained by Vansteenkiste et al. (2009), while the fourth cluster is different. It is possible to note a first cluster of students with a prevalence of controlled motivation, this is the ‘poor quality motivation’ cluster in the Vansteenkiste et al. (2009) paper. A second cluster of students with strong autonomous motivation corresponds to the ‘good quality motivation’ cluster. A third cluster is composed of students with low total motivation, the quantity of motivation is low for students in this cluster. The sample in this study does not show a cluster of students with a high quantity of motivation. The fourth cluster is composed of students with a low level of quantity and not very good quality, i.e. prevalence of controlled motivation.

This discrepancy can probably be ascribed to a slight difference in the variables used: while in our definition the variables used to measure the quantity and the quality of motivation are effectively independent, in the work of Vansteenkiste et al. (2009) the variables are strictly correlated. In fact, the authors used linear combination of the item scores in the SR-S to define quality and quantity of motivation. Despite this minor disagreement, it is extremely interesting to highlight how the analysis carried out in this study allows to compare the results of a free-text questionnaire with the outcomes of a structured test, based on strong theoretical assumptions.

## Discussion

The examination of motivations that drive first year undergraduate students to choose a university nursing course has been the focus of the present research.

In the present study, a set of 18 categories was developed. The categories, obtained applying a thematic analysis to self-reported statements about the choice of nursing degree course, are in line with findings in research literature both in term of typology and relative frequencies [17–22].

This research represents a step forward in the direction of a deep interpretation of students’ motivation. Indeed, it proposes a methodology, organizing the categories within the theoretical framework of SDT. The dimensional analysis carried out in this study has shown that nursing students’ motivations can be organized along one dimension, corresponding to the controlled/autonomous pattern of the SDT. The distribution of the categories reproduces a classification in which there are categories related to controlled motivation (JOB, PRA, REC and FAM), and categories related to autonomous motivation (SMI, HEL, EXP and HUM).

In a person-centred context, the estimation of the parameters of the unidimensional model permitted us to obtain motivational profiles for students on a nursing degree course. These profiles were in line with the results of other studies that used scales to evaluate student motivation, and were useful in distinguishing between quality and quantity in personal motivation [33]. Three motivational clusters that are comparable with the clusters obtained by Vansteenkiste et al. (2009), were identified: a) students with good quality motivation profile (high autonomous and low controlled); b) students with a poor quality motivation profile (low autonomous and high controlled); c) students with low quantity motivation profile (low autonomous and low controlled). The fourth identified cluster was different: students with a low level of quantity and not very good quality (i.e. prevalence of controlled motivation).

The use of the SDT framework has allowed to obtain a coherent interpretation of the students answers and has created the possibility to clusterise the nursing students into categories according to their motivational profiles. This has made it possible to compare the results of our study with studies carried out in other academic contexts and using different tools.

The application of the methodology proposed here can permit educators to obtain a more homogeneous vision of the motivations and reasons why students enrol on an academic nursing course. This vision can also help to improve academic policies and learning strategies aimed at dealing with problems related to retention and attrition rates in nursing courses.

Given the international nursing shortage, with nursing student retention acknowledged as a priority, strategic approaches to enhance their retention are essential. Knowing the reasons for choosing nursing studies is important for educators in order to foster and support undergraduate student motivation. The study of motivation in this context is usually aimed at measuring the quantity of motivation. The application of the SDT framework also allow to take into account the quality of personal motivation, in terms of *controlled* vs. *autonomous* motivation.

In different context, some studies show that students with an autonomous profile are more persistent in an academic curriculum than students with other motivational profiles [33, 34]. In addition, intrinsic motivation can also serve to prevent academic disengagement [29]. This knowledge should make it possible to implement effective strategies aimed at reducing attrition and improving academic success. In fact, as SDT argues, the social environment is fundamental in modifying motivation. Interpersonal environments that are autonomy supportive enhance autonomous motivation and promote learning [47]. On the other hand, controlling environments reduce autonomous motivation and increase controlling motivation.

Promoting specific interventions and activities to support both lessons and internships, may encourage or reinforce motivational aspects already present in students that are essential for this typology of study and profession. One modality would be to offer opportunities for consultation and confrontation, activities in which students can discuss, analyse and reflect on their continuous study experiences and internships that are provided within the nursing course. Another approach would be to make lessons more interactive, with the use of simulation activities, workshops, discussions, reflections, etc. These activities may facilitate the creation of educational environments that allow students to satisfy their basic needs for autonomy, competence and relatedness, and contribute to retention in nursing studies. Further research is needed to evaluate the efficacy of these autonomous supportive interventions, in terms of modification or preservation of the good quality of motivations.

## Limitation and future research

This study has some limitations. To validate the results of this study, further studies in other social and cultural contexts, using also other instruments instead of open questions, such as multiple choice questions, structured interviews, focus groups, etc., are needed.

This study represent a first step to better understand the association between the motivational profile of nursing students and learning success and rate of attrition. In fact, intrinsic motivation is expected to be associated with more successful student learning outcomes and success over time, than controlled motivation. Furthermore, intrinsically motivated students will probably persist much more in academic tasks. To investigate the validity of these results following the students along their academic career may be indicated.

The cross-sectional nature of this study did not permit to obtain causal relationships between motivations and other situational or dispositional individual traits, such as test anxiety [48]. Only a longitudinal study can help to better understand the evolution of individual motivations and the richer dynamic of interactions between personal variables and other characteristics of the context. Moreover, a wider comparison is needed to verify the validity of the procedure proposed and to improve the description of the possible ‘motivational landscapes’ which are most favourable to the academic success of nursing students.

Another possible further advance of the present study is the opportunity to develop a new quantitative survey, that could be administered to students after their admission to the degree course, which would investigate well known motivational factors for choosing a nursing career within the framework of SDT.

Despite the above limitations, this study offers initial empirically based evidence of a common motivational pattern characterising students on a nursing degree course. The SDT appears to be a good theoretical framework to describe and analyse this pattern, independently of individual and contextual differences.

## Conclusion

This study analysed within the theoretical framework of SDT the answers to open questions aimed at investigating the self-reported motivations of nursing students in the first year of their degree course. It proposed a methodology to overcome some of the biases typical of self-reported data, integrating the qualitative analysis of the nursing students’ statements with multi-dimensional scaling techniques aimed at interpreting the results of the qualitative analysis in the framework of SDT.

For this purpose, we analysed the student nurses’ answers using both qualitative and quantitative methods. The qualitative analysis allowed us to collect a large quantity of data about study choices. The aim of this analysis was to identify the main reasons students indicate to motivate their choice of the nursing course. A set of 18 categories was developed. The quantitative analysis utilised the SDT framework to organize these categories along a continuum ranging from controlled to autonomous motivation.

Through adoption of a person-centred approach, four motivational profiles were identified: a) students with good quality motivation profile; b) students with poor quality motivation profile; c) students with low quantity motivation profile; d) students with low quantity and poor quality motivation profile.

The significance of this study includes the opportunity to understand nursing students’ reasons within the theoretical framework of SDT, a well-grounded model able to offer useful information to educators, in order to plan effective strategies to foster student motivation, to enhance student retention and successful outcomes, to prevent student attrition and prepare students to enter the workforce.

## Abbreviations

SDT: Self-determination theory
CFA: Confirmatory factor analysis
FA: Factor analysis
